# Enhanced photocatalytic degradation of methylene blue dye using eco-friendly synthesized rGO@ZnO nanocomposites

**DOI:** 10.1038/s41598-023-48826-7

**Published:** 2023-12-14

**Authors:** Asfaw Negash, Said Mohammed, Hulugirgesh Degefu Weldekirstos, Abera D. Ambaye, Minbale Gashu

**Affiliations:** 1 Department of Chemistry, Debre Berhan University, P.O. Box 445, Debre Berhan, Ethiopia; 2 Institute for Nanotechnology and Water Sustainability, University of South Africa, Florida Science Campus, Johannesburg, 1710 South Africa; 3 Materials Science and Engineering Research, Bio and Emerging Technology Institute, P.O. Box 5954, Addis Ababa, Ethiopia

**Keywords:** Environmental sciences, Chemistry, Materials science, Nanoscience and technology

## Abstract

Industrial chemical pollutants such as methylene blue (MB) dye are released into the water body and potentially cause harm to the human and aquatic biosphere. Therefore, this study aims to synthesize eco-friendly nanocatalysts, i.e., reduced graphene oxide (rGO), zinc oxide (ZnO), and reduced graphene oxide-zinc oxide (rGO@ZnO) nanocomposites, for efficient photocatalytic degradation of MB dye. A graphite rod was obtained from waste dry cell batteries for the electrochemical exfoliation synthesis of graphene oxide (GO) and rGO. For the eco-friendly synthesis of ZnO and rGO@ZnO nanocatalysts, Croton macrostachyus leaf extract was used as a reducing and capping agent. The synthesized nanocatalysts were characterized using a UV–Vis spectrophotometer, Fourier transform infrared spectroscopy, X-ray diffraction, and scanning electron microscopy with energy-dispersive X-ray. The eco-friendly synthesized rGO, ZnO, and rGO@ZnO nanocatalysts were applied for the photocatalytic degradation of MB dye using direct sunlight irradiation. At optimum parameters, photocatalytic degradation of MB dye efficiency reached up to 66%, 96.5%, and 99.0%, respectively. Furthermore, kinetics of the photodegradation reaction based on rGO, ZnO, and rGO@ZnO nanocatalysts follow pseudo-first-order with a rate constant of 2.16 × 10^–3^ min^−1^, 4.97 × 10^−3^ min^−1^, and 5.03 × 10^−3^ min^−1^, respectively. Lastly, this study promotes a low catalyst load (20 mg) for the efficient photodegradation of MB dye.

## Introduction

In the twenty-first century, the impact of environmental pollution stemming from natural and anthropogenic activities has emerged as a critical global issue posing a formidable challenge to both human and aquatic life^1,2^. The complex interplay of various factors contributing to this environmental degradation has intensified the urgency of addressing this problem with concerted efforts towards sustainable practices and mitigation strategies^3^. The industrial manufacturing procedures are consuming a substantial quantity of potable water in their operations^4^. Concurrently, industries discharge a significant volume of the dye wastewater into various water reservoirs^5,6^. The discharged wastes contain different classes of dyes such as basic, acidic, disperse, mordant, reactive, and ingrain azo dyes. These industrial pollutant dyes directly or indirectly, and adversely affect the well-functioning of the ecosystem like water bodies^7^.

Removal of dyes from industrial wastewater is a challenging issue due to their characteristic properties, non-degradable and persistent nature^8^. Amongst them, MB dye is one of the water soluble cationic thiazine dyes disposed frequently to the environment by industrial activities^9,10^. It is poisonous, carcinogenic, and non-biodegradable; it poses a major risk to public health and has a negative impact on the ecosystem. It is commonly released in huge amounts in water bodies by most of the textile industries. To ensure the environmental norms, the toxic materials should be degraded into environmentally friendly materials before their disposal. However various technologies such as chemical ozonisation^11^, ion exchange^12^, adsorption^13^, and coagulation/flocculation^14,15^ have been studied to remove such toxic pollutants from wastewater^15^. Nevertheless, these techniques are not proficient to remediation of all contaminants from wastewater or produce a high concentration of pollutant sludge wastes.

To this end, photocatalysis technology have attracted much attention for the wastewater treatment applications using UV–Vis light irradation^16^. The vast benefits of photocatalysis processes over other methods to eliminate pollutants from wastewater are the consumption of low cost renewable energy. Among the different photocatalysis, recently nanoscience and nanotechnology have transformed the entire biosphere^17^. Nanomaterials such as ZnO exhibits the hexagonal wurtzite crystal structure and n-type semiconductor with band gap of 3.3 eV, and has been applied for many devices such as ultrasonic transducers, and oxygen sensor^18^. Recently, researchers integrated the application of nanomaterials towards photocatalyst under Uv–Vis irradiation^19^. Among the different nanomaterials ZnO takes the attention of researchers as non-toxic treatment method of wastewater polluted by dyes like MB using UV–Vis light irradation^14^. When a suitable catalyst induces, the dye adsorbs on the active site of the catalyst, and in turn converts it to carbon dioxide and water in the presence of Uv–Vis radiation. The performance of the photocatalytical activity of nanoparticles (NPs) is very dependent on the synthesis method and the starting precursor materials^20^. There are several synthesis methods and reagents for regulating the particle size and shape at the nanophase. For example, the photo-catalytic activity of ZnO-NPs synthesized by precipitation and sol–gel method applied for the photodegradation of MB dye scored up to efficiencies 81% and 86%, respectively^21^. On the other hand, ZnO synthesized by hydrothermal methods removed up to 94% of the MB dye pollutants in 80 min^22^. However, to obtain the appropriate size and nanophase structure of the nanosynthesis, there are still experimental bottlenecks^23^. To this end, a new synthesis method has been reported recently using plant extract as a potential capping and stabilizing agent as well as a binding agent for nanocomposite formation. For example, the green synthesized ZnO-NPs using extracts of *Justicia spicigera* degraded 90% of the MB dye photocatalytically^24^. Nonetheless, the electron–hole recombination ZnO-NPs photogenerated the as-cast photocatalysts is too fast, and using the solar spectrum in the visible range is inefficient. It minimizes photocatalytic ability and significantly restricts commercial applications. Researchers use the methods to improve the energy efficiency of photocatalysts performance of semiconductor catalysts composite nanostructures by combining with carbon nanostructured materials^25,26^.

The carbon-based nanostructured material like graphene has its own unique properties, such as being a conductive carrier, adsorbent, photosensitizer, photostabilizer, photocatalyst, and co-catalyst in nanocomposites^27^. The most prominent synthesis methods for graphene are Hummer’s method and its modified versions^28^. GO is a highly oxygenated form of graphene^29^. However, rGO is an alternative form of graphene because it has lower functional groups than GO. Moreover, due to its high electron conductivity, rGO serves as a carrier for supporting materials in the photocatalytic process. Likewise, the rGO and metal oxide NP composite materials exhibit stronger photocatalytic abilities than pure metal oxide. The photocatalytic performance of the ZnO semiconductor was enhanced by incorporating rGO^30^. Recently, the photocatalytical degradation of MB dye using rGO@ZnO nanocomposite showed improved photocatalytic performance^31^.

In order to prepare rGO, electro-reduction methods have recently emerged as modest and effective alternatives for chemical reduction of GO. Electro-reduction of GO has revealed substantial benefits, such as low cost, easy operation, and environmental friendliness. However, for the synthesis of rGO, most researchers used commercial graphite. In this paper, we used waste dry cell batteries as the source of graphite for the electrochemical exfoliation of GO. The rGO, ZnO, and rGO@ZnO nanocomposites have been successfully synthesized using the combined electrochemical exfoliation method, followed by the modified Hummer’s method and the eco-friendly/green synthesis method, respectively. For the green synthesis, ZnO and rGO@ZnO nanocomposite *C. macrostachyus* plant extracts were used as capping and stabilizing agents. The *C. macrostachyus* (family *Euphorbiaceae*) is indigenous in Ethiopia and other Eastern African countries^32,33^. The *C. macrostachyus* plant is found from 1300 to 2500 m above sea level. In Ethiopia, the Shinasha, Agew, and Amhara people use the *C. macrostachyus* plant widely for the traditional medical treatment of malaria^32^. The leaf of the plant is chemically composed of phenolic compounds, flavonoids, saponins, tannins, terpenoids, alkaloids, and cardiac glycosides^32,33^.

Lastly, the eco-friendly synthesized nanocatalysts rGO, ZnO, and rGO@ZnO were applied for MB dye photodegradation under direct sunlight irradiation at optimized parameters. The photodegradation performance of MB dye (20 ppm) using rGO, ZnO, and rGO@ZnO was 66%, 96.5%, and 99%, respectively, with a small catalyst load (20 mg) at 100 min. The findings of the study promote the low catalyst dose load for the fast and efficient photodegradation of MB dyes, and it is a promising output for the low-cost commercialization of photonanocatalysts.

## Materials and methods

### Materials

Analytical-grade chemicals such as zinc acetate dihydrate powder (CH_3_COO)_2_Zn·2H_2_O (99.5%), sodium hydroxide pellets (NaOH, 99.8%), used dry cell batteries, *C. macrostachyus* leaf, and methylene blue (C_16_H_18_ClN_3_S) were used in this study without further purification. Double-distilled water was used throughout the whole experiment.

### Preparation of aqueous extract

The *C. macrostachyus* leaves were collected from the Ankober Botanical Research Centre of Debre Berhan University (DBU). The wild plant in the nursery site was identified, and a voucher specimen was deposited (M4) by a professional botanist (Dr. Abiyou Tilahun) at the Department of Biology (DBU). The fresh leaves were washed with distilled water to remove any dust and dried in a shade. Then the dried leaves of the plant were ground using a grinder. The powder plant (20 g) was boiled in 200 mL of water at 60 °C for 30 min. The resultant aqueous extract was allowed to cool and then filtered with filter paper to produce a yellowish-colored filtrate for the synthesis of ZnO nanostructures.

### Synthesis of ZnO

**Figure 1:**
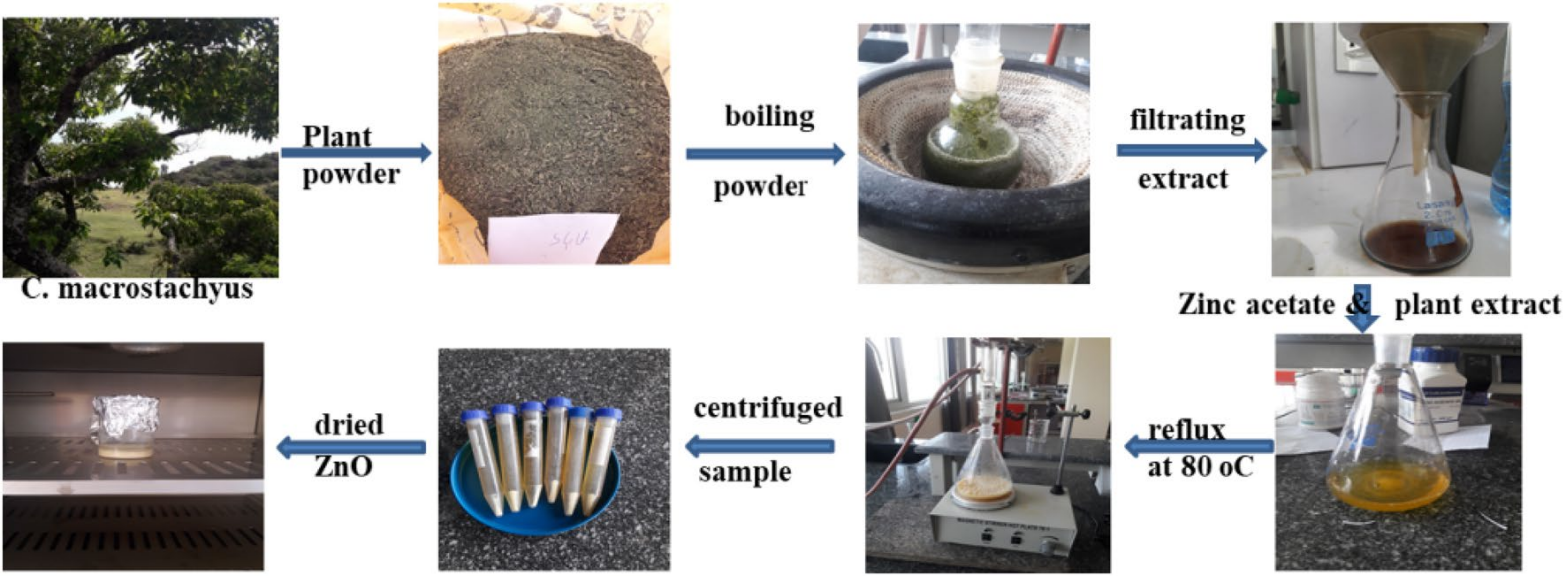
Synthesis route of ZnO.

ZnO nanoparticles were synthesized from 20.0 mL of aqueous plant extract, 0.5 g of zinc acetate dihydrate, 0.2 g of NaOH, and 80 mL of distilled water by refluxing at 60 °C under magnetic stirring for 1 h until precipitate formation (Fig. 1)^34–36^. The ZnO NPs were centrifuged and washed periodically with distilled water in order to remove unreacted precursor, and the white ZnO precipitate was dried using a vacuum oven at 100 °C for 4 h.

### Synthesis of GO and rGO

**Figure 2:**
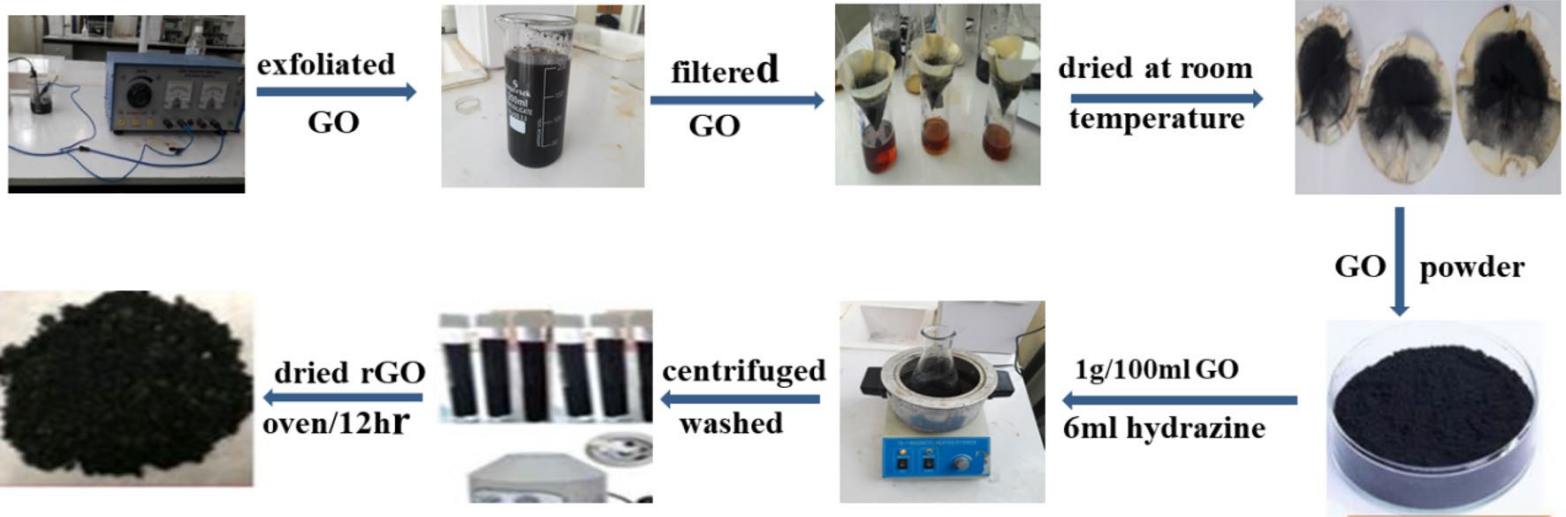
Synthesis route of GO and rGO.

Electrochemical exfoliation experiments were carried out with a two-electrode system^37^. A graphite rod obtained from dry cell batteries is used as an anode electrode and source of graphene, and a platinum wire electrode was employed as a cathode electrode. The experiments were carried out at room temperature by using a low-tension, variable-voltage supply. A constant of 10 V was applied to the electrodes. A sodium hydroxide/water (NaOH/H_2_O) solution was used for efficient electrochemical exfoliation of graphite^37^. The exfoliated GO was washed with deionized water and ethanol several times using filter paper to eliminate the remaining contamination. The cleaned GO powder was dried in a vacuum oven at 60 °C for 24 h. To synthesize rGO from electrochemically exfoliated GO, in an oil bath at 90 °C under continuous stirring for 12 h, the mixture of 6 mL of hydrazine hydrate and 1.0 g of GO was heated in a conical flask containing 100 mL of deionized water (Fig. 2). Finally, to obtain rGO, the black precipitate was centrifuged, eroded repeatedly, and dried at 300 °C for 12 h.

### Synthesis of rGO@ZnO nanocatlysts

**Figure 3:**
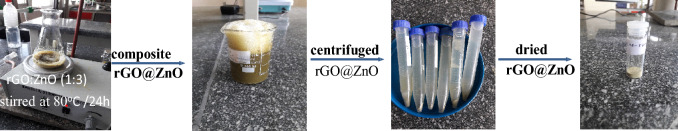
Synthesis route of rGO@rGO-NCs.

20 mL of aqueous extract of *C. macrostachyus* as a reducing and stabilizing agent and 80 mL of distilled water were used for the eco-friendly synthesis of rGO@ZnO-NCs (Fig. 3). The blend ratio of rGO:ZnO was 1:3 based on the literature report by Jana et al*.*^38^. The *C. macrostachyus* plant extract was added dropwise at 60 °C under constant stirring for 12 h. Afterwards, the precipitate was subjected to purification involving the use of both distilled water and ethanol to eliminate any contaminants. Ultimately, the resulting gray precipitate was subjected to drying in an oven, and the formation of the rGO@ZnO NCs was verified via spectroscopic analysis.

### Plant use guideline statement

The collection of plant material and the performance of experimental research on such plants complied with the national guidelines of Ethiopia.

## Results and discussion

### Characterization of synthesized materials

#### Optical properties

**Figure 4:**
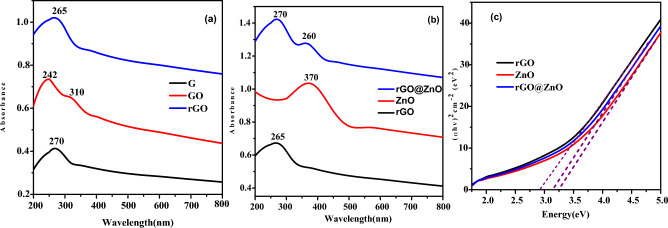
Uv–Vis absorption spectra of (**a**) G, GO and rGO, (**b**) rGO, ZnO, and rGO@ZnO nanocatalysts, and (**c**) corresponding (*αhv*)^2^ versus the *hn* for rGO, ZnO and rGO@ZnO nanocatalysts.

Experiments were conducted using a UV–Visible spectrophotometer to examine the optical characteristics of G, GO, rGO, ZnO, and rGO@ZnO NCs (Fig. 4). G, GO, and rGO were dispersed in deionized water, and G demonstrated an absorption peak near 270 nm. Subsequently, the oxidation of G to GO was ascribed to the occurrence of an absorption peak near 242 nm (Fig. 4a). The occurrence of C=C, C–O, and C=O is attributed to the π–π* transition of oxygen-containing carbonaceous bands in GO^39^. In the optical spectrum of GO, the absorption peak of G at 270 nm vanished. In the spectra of rGO, the disappearance of its absorption peak at 310 nm was observed. The red shift of the absorption peak of GO from 242 to 265 nm ascribed to the structure being restored, increased π-electron concentration, and structural ordering (Fig. 4a). It is in accordance with the recovery of sp^2^ carbon and the conceivable reordering of atoms^40^.

A strong absorption peak attributed at 370 nm in the UV–Visible spectra of eco-friendly synthesized ZnO-NPs (Fig. 4b) is consistent with the characteristic absorption spectra of ZnO reported in the literature. In the spectra of rGO@ZnO, the absorption peaks observed at 270 and 360 nm were due to the absorption of rGO and ZnO, respectively (Fig. 4b). The presence and formation of a Zn–O–C bond between rGO and ZnO-NPs are attributed to the absorption peak at 370 nm. Moreover, compared to pristine rGO, a slight red shift from 265 to 270 nm was observed in the absorption peak of rGO@ZnO (Fig. 4b). The blue shift of rGO@ZnO-NCs ascribed the band edge absorption at 360 nm. It is 10 nm less than the bandgap absorption of bulk ZnO at 370 nm. This is probably described by the quantum confinement result of the smaller feature size of ZnO.

Furthermore, to extrapolate the optical bandgap from the plot of (αhν)^2^ versus hν of the eco-friendly synthesized nanocatalysts, the following equation was used (Fig. 4c). Where α is the optical absorption coefficient, K is constant, hν is the incident photon energy, E_g_ is the optical band gap, and n illustrates the type of optical transition. In the present case, n = 1/2 is considered.

$$\alpha hv = K\left( {hv - E_{g} } \right)^{n}$$

The bandgaps of rGO, ZnO, and rGO@ZnO are 2.92, 3.25 eV, and 3.14 eV, respectively (Fig. 4c). The bandgap energy of rGO@ZnO was reduced to 3.14 eV after the incorporation of rGO, mainly due to the transfer of the photogenerated electron from ZnO to rGO^39^.

#### FTIR spectroscopy analysis

**Figure 5:**
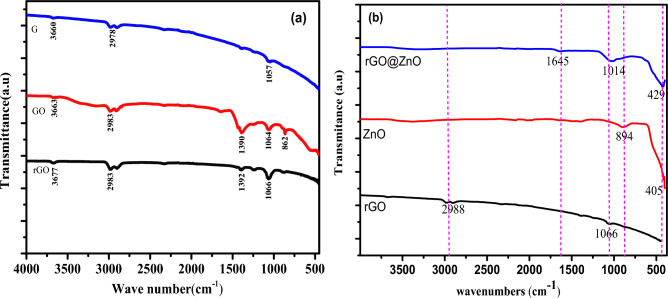
The FTIR spectra of (**a**) G, GO and rGO, and (**b**) rGO, ZnO, and rGO@ZnO.

To investigate the different types of functional groups formed in the powdered G, GO, and rGO, the FTIR spectroscopy experiment was carried out (Fig. 5a). The characteristic band of G was observed at 3660 cm^−1^, 2978 cm^−1^, and 1632 cm^−1^, due to O–H, C–H, and C**=**C stretching vibrations. The electrochemically synthesized GO FTIR spectrum showed a band at 1064 cm^−1^ due to the C–O stretching vibrations. A broad band at 3663 cm^−1^ is ascribed to the O–H stretching vibration of the C–OH groups. The characteristics bands observed at 1390 cm^−1^, and 862 cm^−1^ due to symmetric aliphatic C–H bending of methyl groups, –CH_3_–, and aromatic out-of-plane rings with two neighboring C–H groups, respectively^37^. Furthermore, the absorption bands at 2983 cm^−1^ and 2840 cm^−1^ are attributed to the aliphatic asymmetry of the C–H stretching. The FTIR spectrum of rGO showed the feature bands at 3677 cm^−1^, 2983 cm^−1^, 1392 cm^−1^, and 1066 cm^−1^ due to O–H stretching vibration, C–H stretching vibration, C**=**C stretching, and C–O (alkoxy) stretching. The band strength of the FTIR spectrum of rGO decreased profoundly relative to the band strength of GO. This is a good indication of the reduction and elimination of oxygen comprising functional groups^37^.

Furthermore, the formation of eco-friendly synthesized ZnO-NPs was clearly observed at characteristic absorption bands of 894 cm^−1^ and 429 cm^−1^ when FTIR spectra of ZnO, rGO, and rGO@ZnO were analyzed (Fig. 5b). The FTIR analysis of aqueous *C. macrostachyus* leaf extract assisted for eco-friendly rGO@ZnO NCs demonstrated the absorption bands at 429 cm^−1^, 1014 cm^−1^ and 1645 cm^−1^, representing Zn–O and C–H bending^41^ (Fig. 5b), and C**=**C aromatic configurable vibration may refer to the sp^2^ bonds in the nanostructure, respectively^42,43^. For the details of the FTIR spectra of *C. macrostachyus* leaf extract, see supporting information in Figure S1.

#### X-ray diffraction analysis

**Figure 6:**
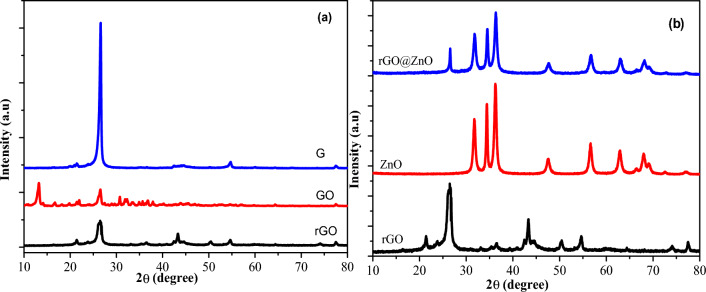
XRD patterns of (**a**) G, GO, and rGO, and (**b**) rGO, ZnO, and rGO@ZnO nanocatalyst.

For phase identification, investigation of the crystallinity and structure of the G, GO, rGO, ZnO, and rGO@ZnO was done (Fig. 6a)^44^. Graphite revealed a sharp and intensive typical peak at 2θ = 26.7°, exhibiting a highly ordered crystal structure with an interlayer spacing of 0.328 nm along with the (002) orientation. The interlayer distance of GO was increased with d-spacing of 0.751 nm, and the peak shifted to 2θ = 10.4°. This is due to the oxidation of graphite into GO. In the XRD spectrum, the inter-layer structure spacing of rGO decreased to 0.369 nm along with the (002) plane orientation. The peak at 2θ = 10.3^o^ disappears, and a new broad peak appears at 2θ = 24.5° due to the decrease of intercalated oxygen functionalities from GO and the restoration of π-conjugated graphene (rGO) (Fig. 6a)^45^. It approves the operative reduction of GO to rGO^39^. Finally, the diffraction peaks of G, GO, and rGO samples were found to be well fitted to the standard XRD pattern of graphite with JCPDS card No. 08–415.

The XRD pattern of the *C. macrostachus* plant extract based eco-friendly synthesized ZnO and rGO@ZnO NCs are shown in Fig. 6b. The peaks for the ZnO NPs at 2θ values of 31.7, 34.4, 36.2, 47.60, 56.6, 62.9, and 67.9° correspond to the planes (100), (002), (101), (102), (110), (103) and (200) according to JCPDS card (36–1451), respectively. The observed high intensity ascribed to the purity and wurtzite hexagonal structure of the ZnO-NPs^46^. The peaks of rGO@ZnO at 2θ values of 26.5, 31.7, 34.5, 36.3, 47.6, 56.7, 62.9, and 68.2° corresponds to the planes (100), (002), (101), (102), (110), (103), (200), and (112), respectively. The peaks of rGO at 2θ values are 21.4, 26.5, 43.3, 50.4, and 54.6°. Distinct peaks related to the rGO were recorded at 2θ = 26.5° in the pattern of rGO@ZnO, which might be due to the crystallinity and 33% w/w amount of rGO in rGO@ZnO NCs (Fig. 6b). Additionally, using the Debye-Scherer equation, the crystallite size of ZnO-NPs was calculated from the most intense peaks^47^. The average crystallite size of ZnO, rGO, and ZnO@rGO is 20.5, 19.9, and 12.5, respectively^48^.

#### Scanning electron microscopy with EDS analysis

**Figure 7:**
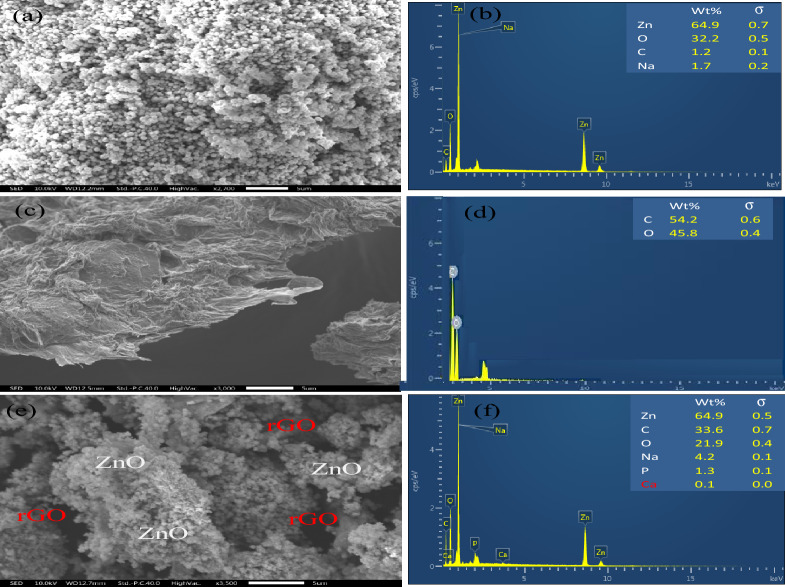
SEM images and corresponding EDS of: (**a** & **b**) ZnO-NPs, (**c** & **d**) rGO, and (**e** & **f**) rGO@ZnO.

SEM was utilized (Fig. 7) to examine the surface morphologies of rGO, ZnO, and rGO@ZnO-NCs. The SEM results indicated that ZnO-NPs exhibited a spherical structure that was evenly distributed (Fig. 7a). The sizes of the ZnO-NPs were found to range from 95 to 105 nm. The SEM image of the rGO synthesized from the electrochemically exfoliated GO exhibited a well-expanded and exfoliated material with a wrinkled or folded thin sheet structure (Fig. 7c). It confirms the successful synthesis of rGO from the waste dry cell batteries. Meanwhile, the rGO@ZnO-NCs size decreased to 15 to 25 nm (Fig. 7e). These findings confirm that the rGO@ZnO-NCs were successfully prepared by utilizing a 33% wt rGO and the C. macrostachus plant extract as a reducing and capping agent. Additionally, to analyze the elemental composition of ZnO, rGO, and rGO@ZnO-NCs, the EDS was carried out (Fig. 7b, d&f). The EDS data corresponding to ZnO and rGO ascribed the anticipated elemental composition of the nanocatalysts. The EDS of rGO@ZnO-NCs also confirms a composition of 39% Zn, 33% C, and 22% O, corresponding to the expected values of elements in rGO@ZnO-NCs. This attributed the rGO@ZnO-NCs successful synthesis using *C. macrostachus* plant extract as a reducing and capping agent. It is also inferred that the rGO was suitably incorporated into the rGO@ZnO-NCs.

### Photocatalytic study

In this study, rGO, ZnO, and rGO@ZnO-NCs were synthesized and used as catalysts for the photodegradation of MB dye under direct sunlight irradiation. The maximum absorbance of MB dye is around 664 nm, which is noted for dye degradation. The initial absorbance was measured prior to irradiation, and the absorbance was measured again at each time interval (t). The photocatalytic degradation performance of rGO, ZnO, and rGO@ZnO NCs was investigated at optimized photocatalytical parameters.

#### Adsorption–desorption equilibrium and the point of zero charge

**Figure 8:**
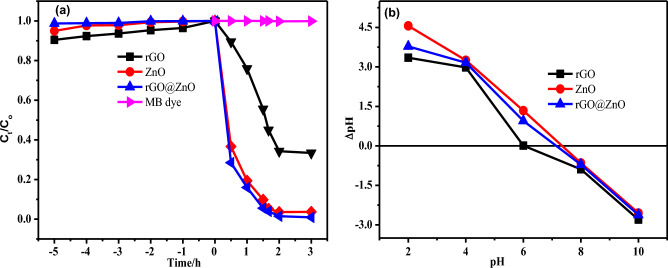
(**a**) Adsorption–desorption equilibrium for 5 h in the dark, and the photodegradation test of the nanocatalysts up to 2 h, and (**b**) The point of zero charge of rGO, ZnO and rGO@ZnO nanocatalysts.

After 5 h, all samples (rGO, ZnO, and rGO@ZnO) reached dark adsorption–desorption equilibrium, as shown in Figure S2 and Fig. 8a. At 5 h, rGO attained the highest adsorption removal of 10%. Furthermore, in the absence of a catalyst, a direct sunlight irradiation experiment was performed on the MB dye solution for 2 h. It resulted in no decrease in solution concentration, indicating that there is no self-photolysis of MB dye.

The point of zero charge for the eco-friendly synthesized rGO, ZnO, and rGO@ZnO-NCs (Fig. 8b) was found to be around 6.0, 7.3, and 7.1, respectively. The surface becomes negative and attracts cations from the solution at pH greater than 6.0, 7.3, and 7.1, and the photocatalytic surface becomes positive and attracts anions from the solution at pH less than 6.0, 7.3, and 7.1, respectively.

#### Effect of pH and photocatalyst dose

**Figure 9:**
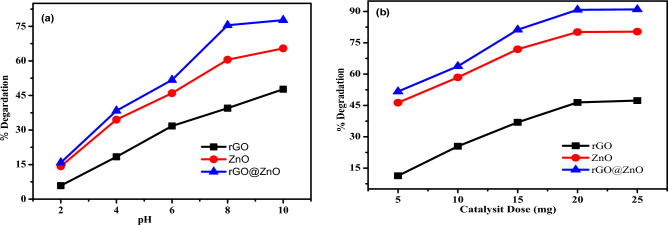
(**a**) pH effect, and (**b**) dosage effect of rGO, ZnO and rGO@Zn of nanocatalysts.

The photo-catalytic degradation phenomena of MB dye with both rGO, ZnO, and rGO@ZnO-NCs increased with increasing pH up to 8, while at low pH (less than 8) and high pH values greater than 8, the photo-degradation performance of MB dye was recorded (Fig. 9a). This is due to the more positive surface charge that might compete with Cl^−^ ions obtained from HCl during pH adjustment. Consequently, uptake of MB cationic dye decreased with lowering pH, thus decreasing its photo-catalytic degradation process. On the contrary, at higher pH, the removal of MB dye decreased due to the excess hydroxyl group from the base, which caused the formation of zinc hydroxide complexes such as Zn(OH)^2–3^, and Zn(OH)^−3^. This effect limited the efficiency and reduced the production of free radicals on the surface of photocatalysts under visible light irradiation. Thus, at a 40 min time span, the maximum photodegradation of MB dye (15 ppm) at pH ≈ 8 was 40.0%, 60.5%, and 76.0% at a fixed catalyst dose of 20 mg for rGO, ZnO, and rGO@ZnO, respectively (Fig. 9a).

The effect of eco-friendly synthesized rGO, ZnO, and rGO@ZnO-NCs photocatalyst doses on the photo-degradation of MB dye was studied by varying its dosage (5 mg, 10 mg, 15 mg, 20 mg, and 25 mg) at a constant volume of 100 mL with an initial concentration of 15 ppm MB dye and at its optimum pH value of 8. The percentage degradation increased when the dose of nanocatalysts increased (Fig. 9b). This is due to the fact that the nanocatalysts provide more surface area absorption sites when they are exposed to visible light irradiation for the production of free radicals. These free radicals also have a greater chance of coming into contact with MB dye. The maximum photo-degradation performance recorded was 47.5%, 80.45%, and 91.58% by rGO, ZnO, and rGO@ZnO, respectively, at 20 mg of the three nanocatalysts (Fig. 9b). With the same amount of nanocatalyst load, rGO@ZnO-NCs recorded better degradation compared to rGO nanosheets and ZnO-NPs. This is due to the presence of an rGO nanosheet in rGO@ZnO, whose energy band gap is lower than that of ZnO^49^ (Fig. 4c). Thus, the rGO@ZnO NCs are more effective than rGO and ZnO for the photo-degradation of MB dye pollutant.

#### Effect of contact time and kinetic study

**Figure 10:**
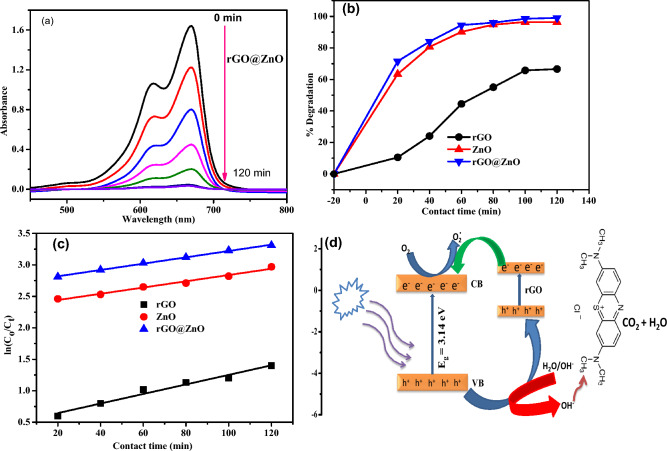
(**a**) UV–Vis spectra of MB photodegradation at different contact times using rGO@ZnO nanocatalyst, (**b**) The effect of contact time on the performance of rGO, ZnO and rGO@ZnO nanocatalyst, (**c**) The first pseudo order kinetics model of photo-degradation of MB dye, and (**d**) the energy level of the photocatalysis process using rGO@ZnO nanocatalyst.

The effect of contact time on the photocatalytic degradation of MB dye was carried out at different contact times (0, 20, 40, 60, 80, 100, and 120 min) at a constant concentration of MB dye (15 ppm), a fixed dose of catalyst (20 mg), and an optimum pH value of 8 (Fig. 10a). The maximum photo-degradation efficiency recorded after 100 min of direct sunlight irradiation was 66%, 96.5%, and 99% for rGO, ZnO, and rGO@ZnO NCs, respectively (Fig. 10b). Additionally, the eco-friendly synthesized rGO@ZnO-NCs catalysts were compared with other previously reported studies on catalysts (Table 1). The study showed the highest performance with a low catalyst dose in 100 mL of 15 ppm of MB concentration. The superior photocatalytic efficiency of rGO@ZnO-NCs over pristine ZnO-NPs is mainly due to photogenerated electrons from ZnO excited by a light source trapped by the reduced graphene oxide, avoiding electron–hole pair recombination^50^. The inset UV–Vis spectra of MB photo-degradation were presented (Fig. 10a) for the most efficient nanocatalyst, rGO@ZnO, as an example. The hydroxy groups can act as adsorption centers for pollutants, which can help enhance the photocatalytic activity of rGO@ZnO-NCs. The presence of oxygen-deficient centers on the surface may slow the recombination of electron–hole pairs. Moreover, the reaction kinetics of MB dye photodegradation in the presence of rGO, ZnO, and rGO@ZnO nanocatalysts were investigated using the following equation (Fig. 10c). Figure (10d) demonstrates the photo degradation mechanism of MB using rGO@ZnO-NCs after being subjected to direct sunlight irradiation.

$$\ln \left( {\frac{{C_{o} }}{{C_{t} }}} \right) = kt$$

**Table 1:** Comparison of the eco-friendly synthesized rGO@ZnO nanocatalysts with other previously reported study.

Nanocatalyst	Light source	Catalyst dose/ mg	MB dye conc./ppm	Irradiation Time (min)	Perfomance/%	Ref
Fe_3_O_4_/rGO/ZnO	Halogen lamp (500 W)	100	100	90	98.5	^52^
rGO/ZnO	UV lamp (100 W)	20	10	165	97%	^53^
CuO/ZnO	visible light	20	20	105	82%	^54^
NiO/ZnO	visible light	75	7 mg/L	175	97%	^55^
rGO-ZnO/CuO	Tungsten halogen lamp (150 W)	20	10	105	90%	^56^
rGO@ZnO	Direct sun light irradiation	20	15	100	99%	This work

The graphs of ln (C_t_/C_o_) were plotted as a function of the reaction time (Fig. 10c). The linearization of the results confirms the first pseudo-order kinetics of the photo-degradation of MB dye. A good correlation to the first pseudo-order reaction kinetics (R^2^ = 0.988 for rGO, R^2^ = 0.992 for ZnO, and R^2^ = 0.998 for rGO@ZnO) was found. The calculated pseudo-first-order rate constants of rGO, ZnO, and rGO@ZnO are 2.16 × 10^−3^ min^−1^, 4.97 × 10^−3^ min^−1^, and 5.03 × 10^−3^ min^−1^, respectively (Fig. 10c). The results further verified that rGO@ZnO is a better photocatalyst for the degradation of MB dye, consistent with the photodegradation performance of the nanocatalysts.

#### Reusability and photostability

**Figure 11:**
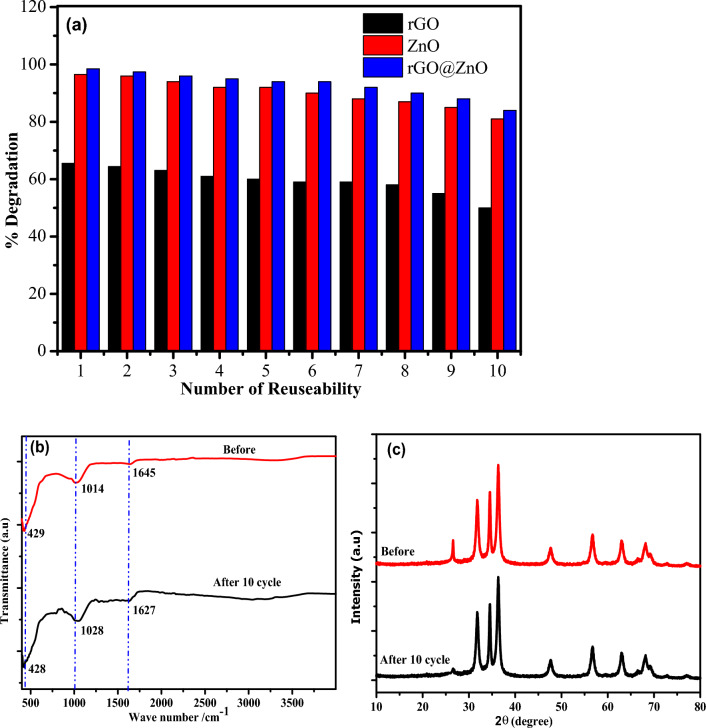
(**a**) The reusability rGO, ZnO and rGO@ZnO, and (**b**, **c**) FTIR, and XRD of rGO@ZnO before and after 10 time’s use of the catalyst.

The reusability and photostability of catalysts are important considerations when selecting a cost-effective and practicable catalyst for large-scale remediation systems. The reusability of rGO, ZnO, and rGO@ZnO-NCs for MB dye photo-degradation was tested ten times using 20 ppm of 100 mL MB dye (Fig. 11a). Under direct sunlight irradiation, the degradation efficiency of MB dye after the tenth run was 50%, 81%, and 84% for rGO, ZnO, and rGO@ZnO, respectively (Fig. 8a). Furthermore, the FTIR spectrum of rGO@ZnO showed a similar bands before and after 10 cycle with a slight shift at 3042 cm^−1^, 1627 cm^−1^, 1047 cm^−1^ and 428 cm^−1^ corresponding to stretching vibration of C–H, C**=**C, C–O and Zn–O, respectively^51^ (Fig. 11b). The XRD spectra showed a similar phase, crystallinity, and structure of rGO@ZnO before and after 10 cycles (Fig. 11c). Hence, both the FTIR and XRD spectra of rGO@ZnO after repeated use confirmed the high photo-stability, which allowed for long-term applications.

## Conclusions

In this study, ZnO-NPs and rGO@ZnO-NCs were successfully synthesized by an eco-friendly method using *C. macrostachyus* leaf extract. The phytochemicals present in the aqueous *C. macrostachyus* leaf extract played a significant role in serving as a capping and reducing agent. The electrochemically exfoliated rGO and eco-frendly synthesized ZnO-NPs and rGO@ZnO-NCs catalysts were characterized using UV–Vis, FTIR, XRD, and SEM–EDS, and the results confirmed the successful synthesis of the nanocatalysts. The synthesized rGO, ZnO-NPs, and rGO@ZnO-NCs catalysts were applied for photocatalytical degradation of MB dye, and their performance was analyzed against the photodegradation of MB dye under direct sun light irradiation. The highest photocatalytical performances recorded for rGO, ZnO, and rGO@ZnO were 66%, 96.5%, and 99%, respectively, at the optimum time of 100 min. Therefore, *C. macrostachyus* leaf extract-assisted synthesized rGO@ZnO-NCs showed promising efficient, eco-friendly, and stable photocatalytic activity with a low catalyst dose (20 mg) for the photodegradation of MB dye.

### Supplementary Information

Supplementary Information.

## Data Availability

Data is available from the author (AN) up on reasonable request.
