# Supplementary material for: Enhanced photocatalytic degradation of methylene blue dye using eco-friendly synthesized rGO@ZnO nanocomposites
Source: Sci Rep. 2023 Dec 14;13:22234. doi: 10.1038/s41598-023-48826-7 (PMC10721910; doi:10.1038/s41598-023-48826-7)
Supplement: Supplementary file 1 — Supplementary Information. [file 41598_2023_48826_MOESM1_ESM.docx]

**Electronic Supporting Information**

**Enhanced photocatalytic degradation of Methylene Blue dye** **using** **eco-friendly synthesized rGO@ZnO nanocomposites**

Asfaw Negash^1*^, Said Mohammed^1^, Hulugirgesh Degefu Weldekirstos^1^, Abera D. Ambaye^3, 4^, and Minbale Gashu^1*^

^1^Department of Chemistry, Debre Berhan University, P.O. Box 445, Debre Berhan, Ethiopia. *Corresponding authors E-mail: [minbalegashu@dbu.edu.et](mailto:minbalegashu@dbu.edu.et), or [asfawnegash@dbu.edu.et](mailto:asfawnegash@dbu.edu.et)

^3^Institute for Nanotechnology and Water Sustainability, University of South Africa, Florida Science Campus, Johannesburg 1710, South Africa

^4^Materials Science and Engineering Research, Bio and Emerging Technology Institute, P.O.Box 5954, Addis Ababa, Ethiopia.

Figure S1. FTIR spectra C. *macrostachyus* leaf extract.

Figure S2. a-c) Adsorption-desorption equilibrium for 5 hours in the dark, and d) The photodegradation test of the nanocatalysts (rGO, ZnO and rGO@ZnO) up to 2 hours.
